# Relationship between motor dysfunction and chewing movement in patients with Parkinson's disease: A transversal study

**DOI:** 10.3389/fneur.2022.1062134

**Published:** 2022-12-09

**Authors:** Taisei Sano, George Umemoto, Shinsuke Fujioka, Yuki Iwashita, Yuriko Dotsu, Naohisa Wada, Yoshio Tsuboi

**Affiliations:** ^1^Swallowing Disorders Center, Fukuoka University Hospital, Fukuoka, Japan; ^2^Department of General Dentistry, Division of Interdisciplinary Dentistry, Faculty of Dental Science, Kyushu University, Fukuoka, Japan; ^3^Department of Neurology, Neuro-Muscular Center, NHO Omuta National Hospital, Fukuoka, Japan; ^4^Department of Neurology, Faculty of Medicine, Fukuoka University, Fukuoka, Japan

**Keywords:** Parkinson's disease, motor dysfunction, chewing movement, tongue pressure, nutritional status

## Abstract

**Objective:**

To assess the impact of chewing movement in patients with Parkinson's disease (PD), we examined the relation between chewing movement and motor dysfunction in association with PD progression.

**Methods:**

Thirty patients with PD (mean age, 68.9 ± 9.0 years; mean Hoehn and Yahr stage, 3.0 ± 0.7) were recruited. The PD condition was assessed in each patient by using the score of Movement Disorder Society Unified PD Rating Scale (MDS-UPDRS) part III score, body mass index (BMI), serum albumin (Alb), and tongue pressure, number of chews, mealtime, and chewing speed were collected. The patients were divided into two groups (mild and moderate PD groups) based on an MDS-UPDRS part III cut-off value of 32.

**Results:**

The chewing speed positively correlated with tongue pressure (*rho* = 0.69, *p* < 0.01) in the mild group, and with BMI (*rho* = 0.54, *p* = 0.03), serum Alb (*rho* = 0.63, *p* = 0.02), and number of chews (*rho* = 0.69, *p* < 0.01) in the moderate group. The MDS-UPDRS part III scores for all participants correlated negatively with chewing speed (*rho* = −0.48, *p* < 0.01), serum Alb (*rho* = −0.49, *p* < 0.01), and positively with mealtime (*rho* = 0.43, *p* = 0.01). Tongue pressure and serum Alb were identified to be as factors affecting the chewing speed (β= 0.560, *p* < 0.01; β= 0.457, *p* < 0.01, respectively).

**Conclusions:**

These results indicated that the progression of motor dysfunction in patients with PD is likely to affect chewing speed and the nutritional status decline may be linked to the impairment of chewing movement in these patients.

## Introduction

Parkinson's disease (PD) is one of the neurological diseases with prominent symptoms including tremors, bradykinesia, muscle rigidity, and postural instability. Dysphagia is also a common symptom experienced by more than 70% of patients with PD (1, 2), and aspiration pneumonia secondary to dysphagia has been determined to be a leading cause of death among patients with PD worldwide (3–6). Dysphagia in patients with PD develops in four phases: oral preparatory, oral propulsive, pharyngeal, and esophageal. The food choking risk increases with the progression of PD; thus, assessment is important during the oral phase, as well as during the pharyngeal phase. This is often accomplished using a videofluoroscopic swallowing study (VFSS) and the flexible endoscopic evaluation of swallowing (FEES). Dysfunction in the oral phase includes tongue and chewing movement impairment, drooling, and limited movement of the mandible (7–11). A study in 2017 investigated chewing function and mandibular movement in patients with PD (12), but studies examining the association between chewing movement and motor dysfunction in association with PD progression remain limited. The previous study examined the chewing function and mandibular movement in a PD group and compared its findings with a control group. As per the results, chewing functions, such as chewing speed and the maximum bite force of patients, were noted to deteriorate with PD (12). In this study, we analyzed which factors correlate with chewing speed and motor dysfunction in patients with PD to examine the relationship between chewing movement and motor dysfunction.

## Patients and methods

### Selection of patients

From September 2019 to March 2021, the Department of Neurology, Fukuoka University Hospital, accepted 101 inpatients who had been diagnosed with PD, had received medication treatment and had undergone VFSS. Severely compromised patients who had received assistance with meals and undergone tube feeding, patients with other neurologic disorders, neuropsychological dysfunction, or head or neck cancers, and those without consent were excluded. Thirty patients recruited were able to effectively communicate with investigators and eat meals by themselves. We established the sample size based on the minimum number required for calculating the correlation coefficient. The patients were divided into two groups (mild and moderate PD groups) based on an MDS-UPDRS part III cut-off value of 32 (13). The two groups were thereafter compared; the correlation between chewing movement and the other investigation items was evaluated, and the factors which might affect chewing movement were analyzed. Additionally, nine inpatients with parkinsonian symptoms (PS), including two patients with progressive supranuclear palsy (PSP) and seven patients with multiple system atrophy (MSA), were recruited around the same time to compare with the 30 patients with PD. All seven patients with MSA were diagnosed with MSA-cerebellar subtype. The PS patients met the same study requirements as the patients with PD. This study was approved by the Ethics Committee of Fukuoka University Hospital (approval number: H20-03-005), and written informed consent was obtained from all participants on the survey days.

### Factors to analyze

**Figure 1:**
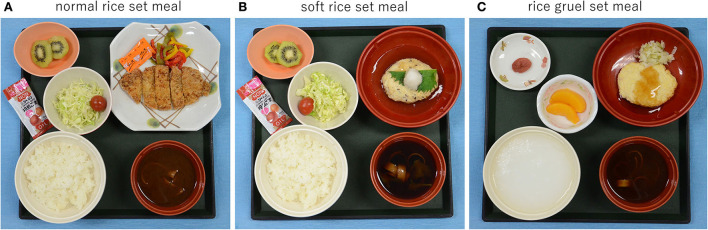
Japanese set meals. **(A)** Normal rice set meal. **(B)** Soft rice set meal. **(C)** Rice gruel set meal.

The mild and moderate PD groups were thereafter compared; the correlation between chewing movement and the other investigation items was evaluated, and the factors which might affect chewing movement were analyzed. They were confirmed to keep bilateral occlusal contacts in the premolar and molar regions of their existing teeth or dentures using the Eichner index. Data including the Hoehn and Yahr stage, the score of Movement Disorder Society Unified PD Rating Scale (MDS-UPDRS) part III score, Mini-Mental State Examination (MMSE) and functional oral intake scale (FOIS) scores, body mass index (BMI), disease duration, serum albumin (Alb), tongue pressure (JM-TPM; JMS, Hiroshima, Japan), number of existing teeth, levodopa equivalent daily dose (LEDD), and mealtime, number of chews and chewing speed (Bitescan^®^ Sharp, Osaka, Japan) were collected. The patients were eating Japanese normal rice set meal, soft rice set meal, or rice gruel set meal which are comparable to 5–7 of FOIS score, respectively (Figures 1A–C). A serum Alb level of < 3.5 g/dL was used as an indicator of malnutrition. Tongue pressure and chewing movement were measured in an “on” state.

### Measurement of tongue strength and chewing movement

Tongue pressure was measured using a handy probe (14). The probe was assembled with a small balloon and pressurized with ambient air to 19.6 kPa. Participants were then asked to compress the balloon against their palate for approximately 7 s using maximum voluntary effort of the tongue. An increase in the inner pressure of the balloon was measured as tongue pressure. This test was repeated three times, and mean values were thereafter obtained.

**Figure 2:**
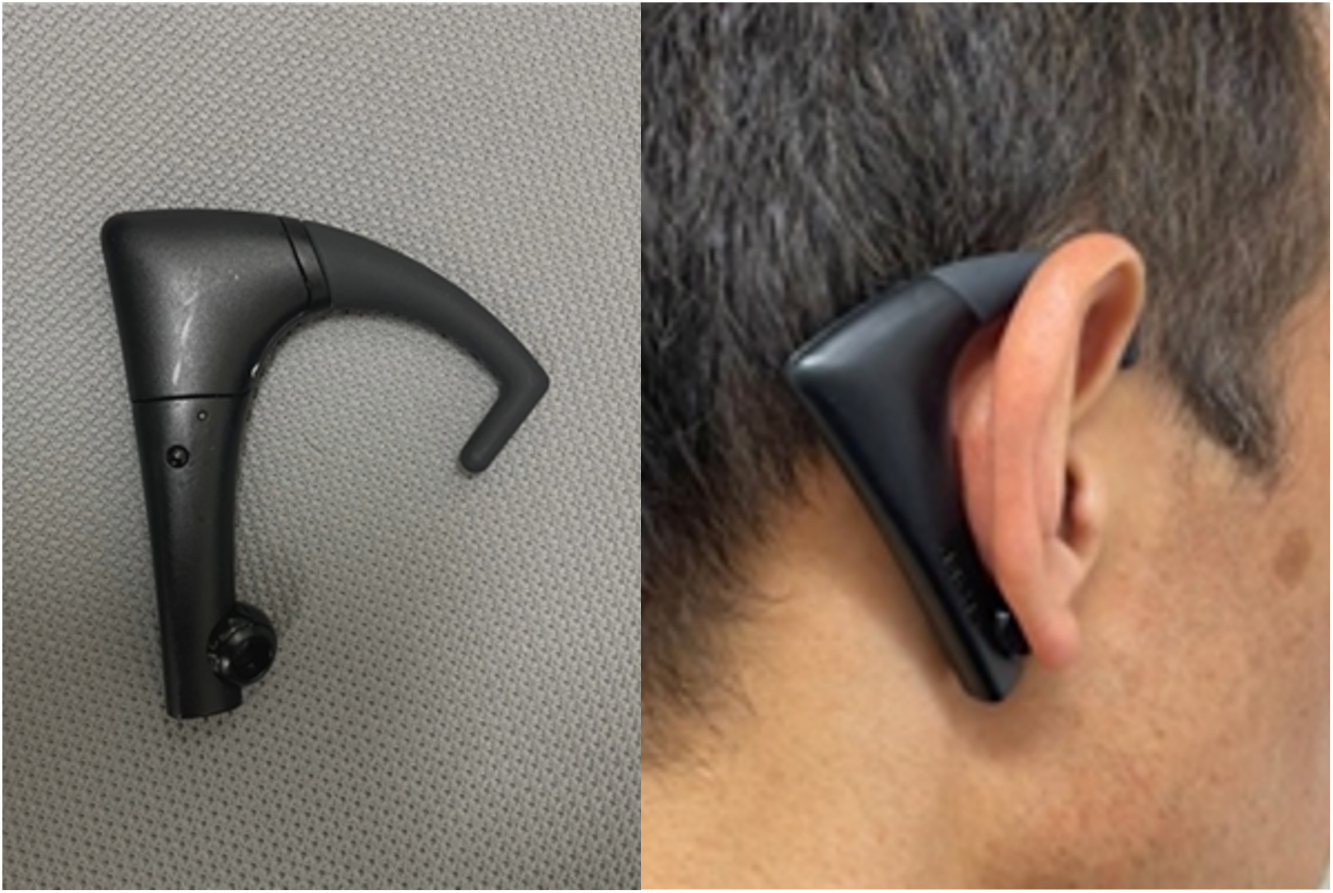
Bite scan^®^ to measure the number of chews, mealtime, and chewing speed.

The chewing movement was measured using a waveform detected on the back of the ear (Figure 2). The number of chews, elapsed mealtime, and chewing speed were measured using the Bitescan^®^ (15). This wearable device has an infrared distance sensor and accelerometer and scans the morphological change in the skin surface on the posterior side of the pinna during chewing exercise, at 20 Hz. This device is designed to be worn on the right side and has an adjustable ear hook (Figure 2). The participants wore the Bitescan^®^ on the back of the pinna, and it was wirelessly linked to a smartphone (SHM05; Sharp Co., Sakai, Japan) *via* Bluetooth. The Bitescan^®^ identified chewing movement from the algorithm installed on the smartphone application which determines chewing based on an individually calibrated threshold of distance, duration, and elapsed time from the last chew, and collected number of chews (15). Chewing speed was calculated by dividing the number of chews by mealtime. The measurement process using the Bitescan^®^ for counting the number of chewing cycles already showed good reliability in a previous study (15).

### Data analysis

Each mean value obtained was compared among groups using a *t*-test, but if the data were not normally distributed, the Mann–Whitney U-test was used for BMI, number of existing teeth, Hoehn and Yahr, mealtime, chewing speed, disease duration, and LEDD instead of a *t*-test. The relationships between chewing speed and all other values of each group, and between motor function and all other values of the PD group were assessed by calculating Spearman's rank correlation coefficient. Multiple linear regression analyses were used to investigate various variables' impact on chewing speed. All statistical analyses were performed using IBM SPSS Statistics for Windows, version 27.0 (IBM Japan, Tokyo, Japan). For all tests, *p* < 0.05 (two-tailed) was considered to be statistically significant.

## Results

There were significant differences in the median age, the number of existing teeth, MMSE, Hoehn and Yahr stage, MDS-UPDRS part III, serum Alb, and chewing speed between the mild and moderate groups of PD (Table 1). There were one and two patients in the moderate groups of PD and the group of PS, respectively, who scored ≤ 21 of MMSE, and two patients in the moderate groups of PD who recorded serum Alb levels < 3.5 g/dL. As per our findings, the moderate group showed significantly higher mean age and value for the Hoehn and Yahr stage (*p* < 0.01, *p* = 0.03, respectively) and lower mean values of MMSE, serum Alb, and chewing speed (*p* = 0.02, *p* = 0.01, *p* = 0.04, respectively).

**Table 1:** Characteristics of participants with Parkinson's disease and parkinsonian symptoms.

	**All participants with parkinsonian symptoms (95% CI)**	**All participants with Parkinson's disease (95% CI)**	***p***	**Mild group of Parkinson's disease (95% CI)**	**Moderate group of Parkinson's disease (95% CI)**	***p***
n	9	30		15	15	
Age(years)	69 (57, 71)	69.5 (65, 72.8)	0.21	65 (61, 68.5)	72 (70.3, 79.3)	<0.01
Sex (m, f)	4, 5	15, 15		9, 6	6, 9	
BMI (kg/m^2^)	23.3 (22.0, 23.7)	20.8 (17.8, 23.9)	0.28	21.6 (18.4, 24.3)	20.8 (17.0, 22.1)	0.31
Number of existing teeth	26 (21, 27)	25 (22, 28)	0.83	27 (23.5, 28)	23.5 (19, 25.8)	0.04
MMSE	27 (22, 28)	27.5 (26, 29)	0.07	29 (27.5, 29.5)	26 (23.5, 28.5)	0.02
Hoehn and Yahr stage		3 (3, 3)		3 (2, 3)	3 (3, 4)	0.03
MDS-UPDRS part III		30 (18.3, 39.8)		18 (12.5, 21)	40.5 (36, 46.8)	<0.01
Serum albumin (g/dL)	4.2 (3.9, 4.2)	4.1 (3.9, 4.3)	0.70	4.2 (4.1, 4.3)	3.9 (3.7, 4.1)	0.01
FOIS	7 (7, 7)	7 (6, 7)	0.06	7 (7, 7)	7 (6, 7)	0.24
Tongue pressure (kPa)	24.1 (22.6, 29.6)	31.1 (24.0, 37.2)	0.20	32.2 (26.5, 37.8)	30.7 (24.8, 32.5)	0.49
Number of chews (times)	726 (415, 808)	833.5 (612.8, 1245.8)	0.19	845 (648, 1216)	873.5 (559.5, 1341.5)	0.76
Mealtime (min)	11.5 (7.8, 13.4)	17.0 (12.8, 20.6)	0.35	16 (11.3, 18.0)	19.9 (13.5, 23.2)	0.08
Chewing speed (times/min)	70.4 (66.5, 76.2)	75.0 (63.3, 83.4)	0.30	77.0 (71.5, 86.6)	66.9 (58.4, 80.8)	0.04
Disease duration (years)	3 (2, 4)	10 (5, 14.8)	<0.01	9 (6, 14.5)	9.5 (4.3, 14.5)	0.61
LEDD (mg)		566 (300, 773.5)		532 (350, 749)	499.5 (300, 764.8)	0.77

**Figure 3:**
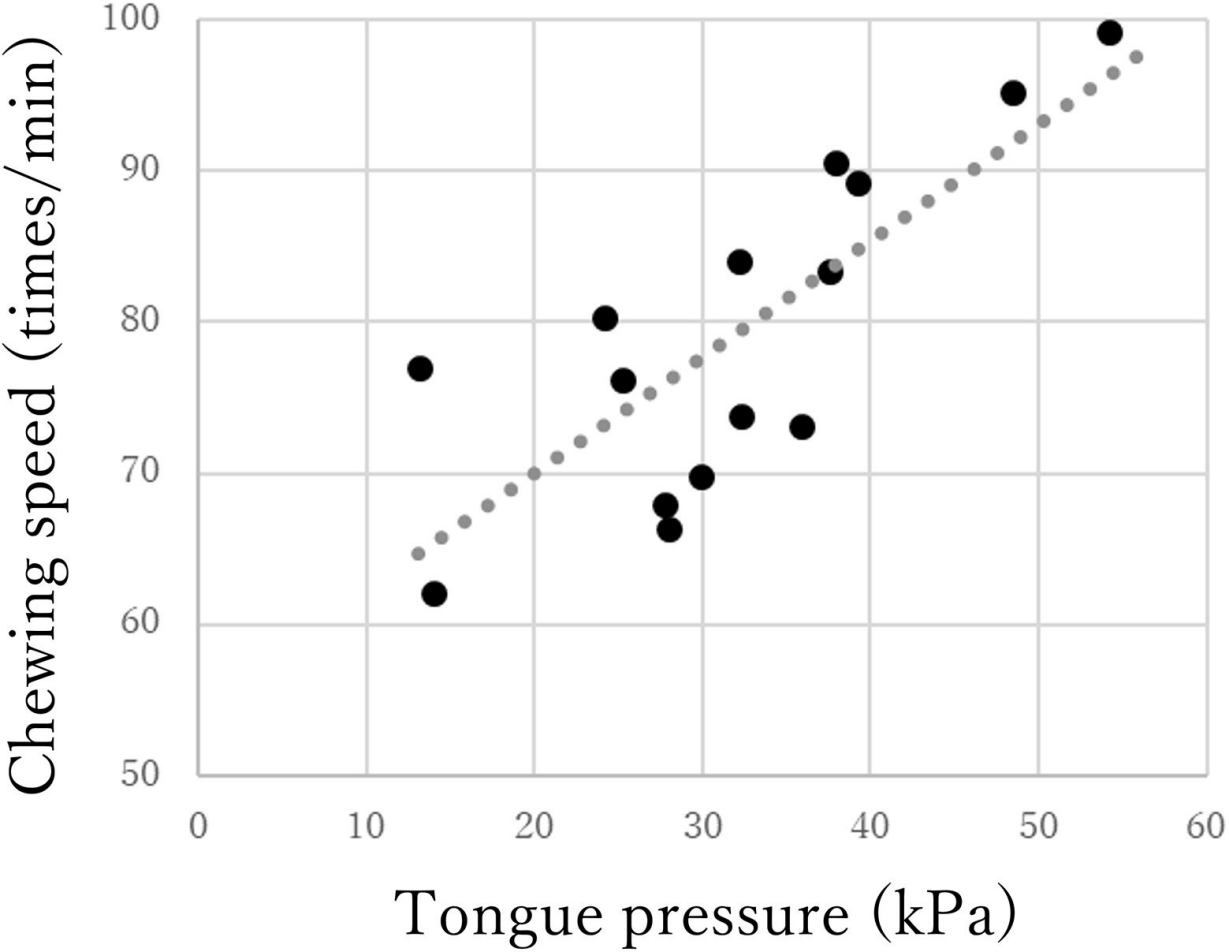
Correlations between chewing speed and tongue pressure in the mild group of Parkinson's disease.

**Figure 4:**
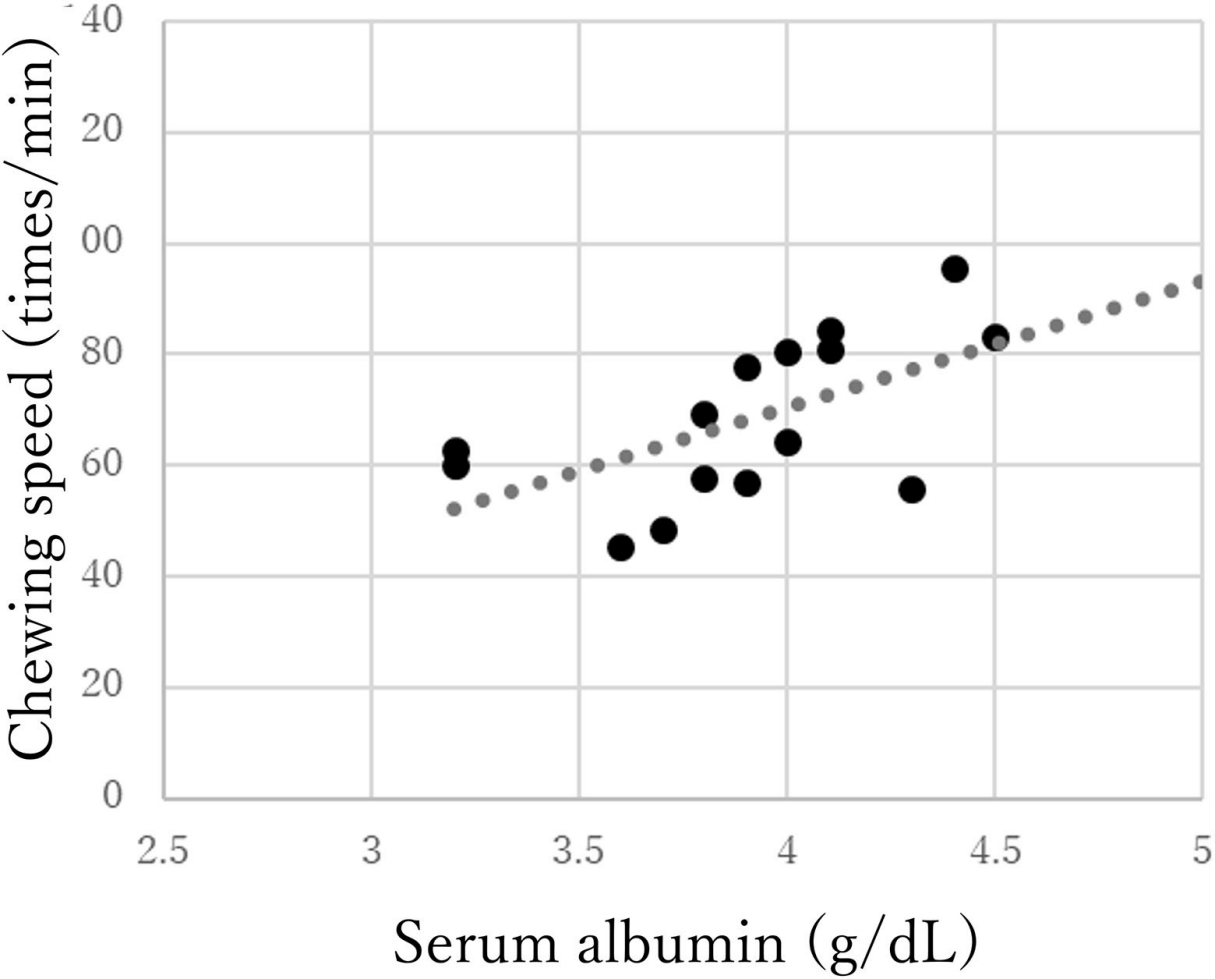
Correlations between chewing speed and serum albumin in the moderate group of Parkinson's disease.

The chewing speed in the mild group positively correlated only with tongue pressure (*rho* = 0.69, *p* < 0.01, Figure 3). The chewing speed in the moderate group showed significant positive correlations with BMI (*rho* = 0.54, *p* = 0.03), number of existing teeth (*rho* = 0.56, *p* = 0.02), serum Alb (*rho* = 0.63, *p* = 0.01, Figure 4), and number of chews (*rho* = 0.69, *p* < 0.01), but not with tongue pressure (*rho* = 0.25, *p* = 0.35) (Table 2).

**Table 2:** Correlations between chewing speed and other factors in mild and moderate groups of Parkinson's disease.

	**Mild group**		**Moderate group**	
	**Spearman's** ***rho***	***p***	**Spearman's** ***rho***	***p***
Age(years)	−0.38	0.15	−0.08	0.77
BMI (kg/m^2^)	0.23	0.40	0.54	0.03
Number of existing teeth	−0.06	0.83	0.56	0.02
MMSE	0.14	0.61	0.35	0.20
MDS-UPDRS part III	−0.44	0.09	−0.33	0.22
Serum albumin (g/dL)	−0.14	0.60	0.63	0.01
Tongue pressure (kPa)	0.69	<0.01	0.25	0.35
Number of chews (times)	0.49	0.06	0.69	<0.01
Mealtime (min)	0.06	0.81	0.06	0.82
Disease duration (years)	−0.28	0.30	−0.19	0.46
LEDD (mg)	−0.01	0.96	−0.07	0.78

The MDS-UPDRS part III scores for all participants negatively correlated with chewing speed (*rho* = −0.48, *p* < 0.01), serum Alb (*rho* = −0.49, *p* < 0.01), and MMSE (*rho* = −0.44, *p* = 0.01) and positively correlated with age (*rho* = 0.66, *p* < 0.01) and mealtime (*rho* = 0.43, *p* = 0.01) (Table 3).

**Table 3:** Correlations between MDS-UPDRS part III scores and other factors of participants with Parkinson's disease.

	**Spearman's *rho***	***p***
Age(years)	0.66	<0.01
BMI (kg/m^2^)	−0.20	0.28
Number of existing teeth	−0.32	0.08
MMSE	−0.44	0.01
Serum albumin (g/dL)	−0.49	<0.01
Tongue pressure (kPa)	−0.13	0.47
Number of chews (times)	0.02	0.91
Mealtime (min)	0.43	0.01
Chewing speed (times/min)	−0.48	<0.01
Disease duration (years)	0.11	0.54
LEDD (mg)	−0.08	0.65

Age, BMI, MMSE, Hoehn and Yahr, MDS-UPDRS part III, serum Alb, FOIS, mealtime, disease duration, and LEDD were included in the stepwise multiple linear regression. The results indicated that the model could explain 50.6% of the chewing speed (*R*^2^ = 0.506). Tongue pressure (β = 0.560, p < 0.001) and serum Alb (β = 0.457, *p* = 0.002) were the influence factors of chewing speed (Table 4). The goodness of fit was assessed to be good enough because of the significant results of ANOVA and the value of R-squared > 0.50.

**Table 4:** Results of multiple regression analysis to examine the effects of factors chewing speed.

	**Unstandardized coefficient β**	**Standardization coefficient β**	***p***	**95%CI**
Constant	−21.651			−6.753~27.724
Tongue pressure (kPa)	0.827	0.560	<0.001	0.417~1.238
Serum albumin (g/dL)	17.238	0.457	0.002	6.753~27.724

R^2^ = 0.506; ANOVA *p* < 0.001.

A significant difference was noted only in disease duration between the patients with PD and PS (*p* < 0.01, Table 1). The patients with PS showed no significant difference in terms of chewing speed compared with the patients with PD, and no significant correlation between chewing speed and certain other factors, in contrast with patients with PD.

## Discussion

The declines in serum Alb and chewing speed in the moderate group were thought to be caused by inadequate nutrition and chewing dysfunction in patients with PD (12, 16). Among the examined factors, tongue pressure was found to have the most impact on chewing speed. Skilled tongue movement and sufficient tongue strength may significantly influence chewing speed. It can be potentially explained by the process model of feeding. The tongue carries food to the post-canine teeth for chewing exercise during stage I transport and propels chewed food into the oropharynx during stage II transport (17–22). The mild and moderate groups showed no significant differences in tongue pressure but in chewing speed. Chewing speed significantly correlated with tongue pressure in the mild group but did not correlate in the moderate group. These findings suggest that the progression of motor dysfunction in patients with PD was more likely to affect chewing speed than tongue pressure. The nutritional status decline associated with the progression of motor dysfunction in PD may be also linked to the impairment of chewing movement. Furthermore, the significant correlation between chewing speed and BMI only in the moderate group suggests that chewing dysfunction could be associated with inadequate nutrition and weight loss as motor dysfunction deteriorates (23).

Weight loss is one of the most common symptoms of patients with PD; it is thought to be caused by decreased energy intake and increased energy expenditure (24). In the face of weight loss in patients with PD, we should pay attention to chewing dysfunction as a possible cause of weight loss, which will secondarily accelerate physical dysfunction or dysautonomia (25). The significant association seen between the progression of motor dysfunction and prolonged mealtimes in this study is consistent with the results of previous research, which suggested the importance of the oral stage of swallowing in patients with PD (7).

The patients with PD showed a significant difference compared with the patients with PS only in disease duration. This may be caused by the narrow inclusion criteria for participation in this study, which limited the patients to those who could communicate and eat by themselves. Generally, the progression rate of PS, including PSP and MSA is faster than PD, and patients with PS usually have oral difficulties within several years of onset due to rapid dysphagic deterioration (26). Parkinsonism is one of the distinctive symptoms in patients with PD and slowing chewing speed showed significant correlations with the deterioration of tongue pressure or nutrition status associated with motor dysfunction. In contrast, the patients with PS did not show such significant correlations because not only parkinsonism but also cerebellar ataxia and/or dysautonomia are easily involved in motor dysfunction.

### Limitations of this study

This study has several limitations. The participants' diet types could not be fully standardized to FOIS levels 5–7 in order to assess their chewing movements. This is because the participants accepted, on demand, the adjustment of their diet types during their various hospitalization periods. We had to conduct this study with a small sample size after recruiting the participants. Furthermore, it is not easy to recruit patients with atypical parkinsonian disorders and eat meals of equal level to patients with PD due to the shorter disease duration of PS. We could not make a simple comparison of patients with PD and PS because of a lack of a standardized assessment tool like the MDS-UPDRS part III. There was no other choice but to judge from the situation that the two groups of patients showed different findings despite they were eating meals of nearly equal levels. In this study, only patients who could eat orally were investigated, and even among these patients, an association between the deterioration of chewing speed and nutrition status was observed. Patients with PD often need adjustment to their diets for nutritional management associated with the progression of dysphagia and PD (27). On the other hand, it was difficult to evaluate how chewing movements were related to adjustments in diet type or the deterioration of dysphagia in relation to the progression of PD. A further study evaluating patients with severe PD using various foods, or in conjunction with an assessment of the pharyngeal stage, will be essential in the future.

## Conclusion

As per the findings of this study, the progression of motor dysfunction in patients with PD is likely to affect chewing movements more than tongue movements. The nutritional status decline associated with the progression of motor dysfunction in PD may be linked to the impairment of chewing movement in these patients.

## Data availability statement

The raw data supporting the conclusions of this article will be made available by the authors, without undue reservation.

## Ethics statement

The studies involving human participants were reviewed and approved by Ethics Committee of Fukuoka University Hospital (approval number: H20-03-005). The patients/participants provided their written informed consent to participate in this study.

## Author contributions

TS and GU were involved in the research project, collecting the data except for the neurological assessments, the statistical analysis, and writing the manuscript. SF, NW, and YT were involved in the neurological assessments, review, and critique of the manuscript. YI and YD were involved in the organization and execution of the research project. All authors read and approved the final manuscript.
